# Paediatric jaw tumours: experiences and findings from a resource limited tertiary health care center

**DOI:** 10.11604/pamj.2020.36.111.23695

**Published:** 2020-06-19

**Authors:** Benjamin Idemudia Akhiwu, Daniel Otasowie Osunde, Helen Oluwadamilola Akhiwu, Ibrahim Aliyu, Kelvin Uchenna Omeje, Basil Ojukwu, Priscilla Okhiabigie Ameh, Rafael Adetokunbo Adebola, Akinola Ladipo Ladeinde

**Affiliations:** 1 Department of Oral and Maxillofacial Surgery, University of Jos, Jos University Teaching Hospital, Jos, Plateau State, Nigeria,; 2 Department of Oral and Maxillofacial Surgery, University of Calabar, University of Calabar Teaching Hospital, Calabar, Cross River State, Nigeria,; 3 Department of Paediatrics, Jos University Teaching Hospital, Jos, Plateau State, Nigeria,; 4 Department of Paediatrics, Bayero University Kano, Aminu Kano Teaching Hospital, Kano, Kano State, Nigeria,; 5 Department of Oral and Maxillofacial Surgery, Bayero University Kano, Aminu Kano Teaching Hospital, Kano, Kano State, Nigeria,; 6 Intercountry Center for Oral Health, Jos, Plateau State, Nigeria,; 7 Department of Preventive Dentistry, University of Jos, Jos University Teaching Hospital, Jos, Plateau State, Nigeria,; 8 Department of Oral and Maxillofacial Surgery, College of Medicine, University of Lagos, Lagos State, Nigeria

**Keywords:** Jaw tumors, paediatric, retrospective study

## Abstract

**Introduction:**

primary maxillofacial tumors are uncommon in pediatric patients. When they do occur, the tissue damage caused directly alters facial growth, development as well as psycho-social evolution. This study was carried out to determine the pattern, sociodemographic characteristics and histologic peculiarities of paediatric jaw tumors in our environment.

**Methods:**

a retrospective hospital-based study where the case notes of children below the age of 14 years who presented with jaw tumors and tumor-like lesions from January 2014 to December 2018 were studied.

**Results:**

eighty-two patients were studied; patients aged 10-14 years had the highest representation. Mean time of presentation was 8 months with jaw swelling being the commonest presentation (84.1%). Majority of the fathers were in their 4^th^ decade of life while most of the mothers were in their third decade of life and both parents possessed primary school certificate as their highest level of educational attainment. Fathers were mostly traders, while mothers were mostly full-time housewives. The maxilla and mandible were most commonly affected with the left side showing higher preponderance. Burkitt lymphoma (19 (23.2%)) and adenomatoid odontogenic tumor (14 (17.1%)) were the commonest lesions. When the tumor involved both the maxilla and the mandible, the tumor was most likely malignant.

**Conclusion:**

in our center, paediatric jaw tumors are commonest in male children with the 10-14 years´ age group most commonly affected. Burkitt lymphoma and adenomatoid odontogenic tumors were the commonest tumors. Early presentation must be encouraged since these tumors if presented early can be successfully treated.

## Introduction

The oro-facial region is a site for multitude of neoplastic conditions [1] leading to broad differential diagnosis [2,3]. Orofacial tumors often cause significant disfiguring effect to facial aesthetics, this may constitute the reason for early hospital presentation. Primary maxillofacial tumors in pediatric patients are not common. Nevertheless, tissue damage caused by these lesions is of greater impact, since, in children, they directly alter facial growth and development as well as psycho-social evolution. Reports of series of jaw tumors in children are rare, thus few oral pathologists have had a wealth of experience in diagnosing these lesions and predicting their biologic behavior. Also, very few surgeons have also documented extensive experience in the management of these lesions. Tumors of the jaw are broadly classified as odontogenic or non-odontogenic depending on their origin. Those originating from teeth forming structures are regarded as odontogenic tumors, while those originating from non-tooth forming structures are regarded as non-odontogenic tumors. However, the odontogenic tumors are said to be commoner in children [4,5]. Most studies carried out in our environment documented the different types of childhood tumors but with little emphasis on the different types of paediatric jaw tumors. Available information suggests that, though paediatric jaw tumors may have a benign histologic appearance, this finding does not frequently predict the aggressive nature of these tumors and there is the need to manage them based on their biologic behaviors and not on descriptive histological diagnosis alone [6]. This study aims to determine the pattern, sociodemographic characteristics and histologic peculiarities of paediatric jaw tumors in our environment.

## Methods

This was a retrospective hospital based study carried out at a tertiary center. The case notes of children below the age of 14 years who presented with jaw tumors and tumor-like lesions from January 2014 to December 2018 were retrieved and studied; patients with missing notes were excluded from the study. Information like patient´s age, gender, educational status and occupation of parents, presenting complaints, duration of symptoms, site of tumor, radiologic findings and histological diagnosis of the tumors amongst others were entered into the relevant aspects of the proforma. Ethical approval was obtained from the ethics committee of the teaching hospital.

**Data analysis:** the data obtained were entered into IBM SPSS statistics for Windows version 22.0 (IBM Corp., Armonk, N.Y., USA) and analyzed. Absolute numbers and simple percentages were used to describe categorical variables. Quantitative variables were described using means (with standard deviation).

## Results

A total of 89 case notes were retrieved. The folders of a total of 82 patients were eventually analyzed (the other seven (7) case notes were excluded because the notes were incomplete). The 82 patients studied were made up of 49 males and 33 females, with a sex ratio (M:F) of 1:1.5. Patients within age-range of 10-14 years constituted the largest group. Majority of both parents of the patients possessed primary school certificate as their highest level of educational attainment and most of the fathers were in the 41-50 years´ age group while majority of the mothers were in the 31-40 years´ age group. Most of the fathers were traders, while most of the mothers were full time housewives. The sociodemographic variables of the patients are shown in Table 1. The commonest presenting complaints was jaw swelling (84.1%) and 22% of the patients studied had missing teeth. The duration of symptoms ranged from less than one month to as much as 4 years. However, majority of patients presented within 1-2 months of onset of symptoms (Table 2). Evaluation of the sites and sides affected by the tumors in the study population showed that the maxilla was the most commonly affected site; this was closely followed by the mandible, while tongue lesions were the least. The cheek, submandibular gland, palate and buccal mucosa, showed similar pattern in their frequency of involvement. The left side showed higher preponderance, while bilateral involvement was only observed in 7.3% of the study population. The site and side of involvement is shown in Table 3. Of the 65 cases involving the mandible and maxilla either singly or collectively, tumors with multilocular radioluscency accounted for 28 (43.1%) of cases and this was followed by unilocular radiographic appearance 21 (32.3%). The other types of radiographic features observed is shown in Table 4. Histopathological analysis of the lesions revealed that Burkitt lymphoma (19 (20.7%)) and adenomatoid odontogenic tumor (14 (14.6%)) were the commonest while pleomorphic adenoma and rhabdomyosarcoma had the least proportions (1.2%). Twenty eight patients had odontogenic tumors while in the other 54, the tumors were non odontogenic (Table 5). The distribution of the maxillary and mandibular tumors as well as whether the tumors were benign or malignant are presented in Table 6. While the age and sex distribution of the tumors are presented in Table 7.

**Table 1:** sociodemographic characteristics of the study population

Variable	N (%)	Mean ± SD
**Age group (Years)**		**8.5 ± 5.2**
0-4	27 (32.9)	
5-9	16 (19.5)	
10-14	39 (47.6)	
**Gender**		
Male	49 (59.8)	
Female	33 (40.2)	
**Fathers age group (Years)**		**42.6 ± 6.2**
31-40	34 (41.5)	
41-50	37 (45.1)	
51-60	11 (13.4)	
**Mothers age group**		**32.0 ± 5.4**
21-30	35 (42.7)	
31-40	46 (56.1)	
41-50	1 (1.2)	
**Occupation of father**		
Trader	33 (40.2)	
Artisan	17 (20.7)	
Civil servant	13 (15.9)	
Farmer	13 (15.9)	
Driver	3 (3.7)	
Cattle rearer	3 (3.7)	
**Mother´ occupation**		
Full-time housewife	79 (96.3)	
Trader	3 (3.7)	
**Fathers education**		
None	3 (3.7)	
Primary	50 (61.0)	
Secondary	9 (11.0)	
Tertiary	4 (4.9)	
Islamic	16 (19.5)	
**Mothers education**		
None	3 (3.7)	
Primary	47 (57.3)	
Secondary	12 (14.6)	
Tertiary	0 (0)	
Islamic	20 (24.4)	
Social class*		
Class 3	4 (4.9)	
Class 4	37(45.1)	
Class 5	41 (50)	

* There were no patients in social class 1 and 2

**Table 2:** common presenting complaints in the patients seen

Complaints	Number (%)	Mean ± SD
Jaw Swelling	69 (84.1)	
Cheek swelling	7 (8.6)	
Swelling inside the mouth	4 (4.9)	
Upper lip swelling	2 (2.4)	
**Missing teeth (n=18)**		
Missing lower canine	3 (16.7)	
Missing premolars	3 (16.7)	
Missing lower molars	3 (16.7)	
Missing upper canine	7 (38.8)	
Missing upper premolars	2 (11.1)	
**Duration of symptoms**		**8.3 ± 10.2**
<1 month	1 (1.2)	
1-2 months	34 (41.5)	
3-4 months	5 (6.1)	
5-6 months	17 (20.7)	
7-8 months	2 (2.4)	
9-10 months	0 (0)	
11-12 months	8 (9.8)	
1-1.5 years	10 (12.2)	
2 years	7 (8.5)	
3 years	4 (4.9)	
4 years	1 (1.2)	

**Table 3:** site and side of the jaw tumor

Variable	Number (%)
**Site of the jaw tumor**	
Maxilla	29 (35.4)
Mandible	22 (26.8)
Maxilla + Mandible	14 (17.1)
Parotid Gland	4 (4.9)
Cheek	3 (3.7)
Palate	3 (3.7)
Buccal mucosa	3 (3.7)
Upper lip	2 (2.4)
Tongue	1 (1.2)
Floor of the mouth	1 (1.2)
**Side of the jaw tumor**	
Left	46 (56.1)
Right	20 (24.4)
Bilateral	6 (7.3)
Midline	10 (12.2)

**Table 4:** radiographic characteristics of paediatric jaw tumors with skeletal involvement (n=65)

Radiographic appearance	N (%)
Unilocular radioluscency	21(32.3)
Multilocular radioluscency	28 (43.1)
Radiopacity	3 (4.6)
Mixed radioluscency/radiopacity	13 (20)
Total	65 (100)

**Table 5:** histopathologic diagnosis of the jaw tumors

Histopathologic diagnosis	N (%)
Burkitt lymphoma (NO)	19 (23.2)
Adenomatoid odontogenic tumor (O)	14 (17.1)
Unicystic ameloblastoma (O)	7 (8.5)
Fibrous dysplasia (NO)	7 (8.5)
Heamangioma (NO)	4 (4.9)
Giant cell granuloma (O)	4 (4.9)
Ossifying Fibroma (O)	3 (3.7)
Adenoid cystic carcinoma (NO)	3 (3.7)
Cherubism (NO)	3 (3.7)
Teratoma (NO)	3 (3.7)
Infantile Fibrosarcoma (NO)	3 (3.7)
Cystic hygroma (NO)	3 (3.7)
Osteoma (NO)	3 (3.7)
Hodgkin´s Lymphoma (NO)	2 (2.4)
Non-Hodgkin´s lymphoma (NO)	2 (2.4)
Rhabdomyosarcoma (NO)	1 (1.2)
Pleomorphic adenoma (NO)	1 (1.2)
Total	82 (100)

O- odontogenic; NO- Non-odontogenic

**Table 6:** distribution of the maxillary and mandibular tumors

Site	Type of lesion	Histopathological diagnosis	N (%)
**Maxilla (n=29)**			
	Malignant	Burkitt lymphoma	10 (34.5)
	Benign	Adenomatoid odontogenic tumor	12 (41.4)
	Benign	Fibrous dysplasia	4 (13.8)
	Benign	Giant cell granuloma	3 (10.3)
**Mandible (n=22)**			
	Benign	Unicystic ameloblastoma	7 (31.8)
	Benign	Cystic hygroma	3 (13.6)
	Benign	Fibrous dysplasia	3 (13.6)
	Benign	Cherubim	3 (13.6)
	Benign	Osteoma	3 (13.6)
	Benign	Ossifying fibroma	3 (13.6)
**Maxilla + mandible (n=14)**			
	Malignant	Burkitt lymphoma	6 (42.9)
	Malignant	Infantile fibrosarcoma	3 (21.4)
	Malignant	Hodgkin´ lymphoma	2 (14.3)
	Benign	Osteoma	2 (14.3)
	Benign	Giant cell granuloma	1 (7.1)

**Table 7:** age and sex distribution of the tumors

Histopathological diagnosis	Age distribution (years)			Sex distribution	
	0-4	5-9	10-14	Male	Female
	n	n	n	n	n
Unicystic ameloblastoma	0	2	5	0	7
Burkitt lymphoma	11	5	3	10	9
Cystic hygroma	3	0	0	0	3
Fibrous dysplasia	0	3	4	3	4
Ossifying fibroma	0	0	3	3	0
Pleomorphic adenoma	0	0	1	1	0
Adenoid cystic adenoma	3	0	0	0	3
Adenomatoid odontogenic tumor	0	0	14	7	7
Rhabdomyosarcoma	0	1	0	0	1
Heamangioma	3	0	1	1	3
Giant cell granuloma	0	1	3	3	1
Cherubism	0	3	0	0	3
Teratoma	3	0	0	0	3
Infantile fibrosarcoma	0	3	0	3	0
Hodgkin´ Lymphoma	0	0	2	0	2
Non- Hodgkin´ lymphoma	2	0	0	0	2
Osteoma	0	0	3	2	1

## Discussion

The exact incidence of paediatric jaw tumors is difficult to estimate owing to the different tumor types and sites included in studies and the different definitions used by authors to imply paediatric age group [7-9]. Our findings showed that children within the second decade of life formed the highest proportion of the subjects with jaw tumors. This period corresponds to the age where there is major transition from deciduous to permanent teeth as well as development of full complement of permanent dentition. This active stage of teeth development forms a crucial period for the development of odontogenic tumors in this age group. Although there were several designated conflicting age ranges considered as constituting paediatric population, majority of the studies favored the second decade as age of highest proportion [10-12]. Our study demonstrated that more males were affected than females this is similar to what was documented by Adebayo *et al*. [13] but is in contrast to the study by Perry *et al*. [7] who documented that females were more affected than males. Saxena *et al*. [4], on the other hand found equal gender distribution. The commonest presenting complaints were jaw swelling which was the presentation in about 84% of the patients with about one in every fourth patient presenting with a missing teeth and a large proportion of the patient presenting within the first two months of onset of symptoms however the mean time of presentation was 8 months. The presentation of a jaw mass is not surprising and was also documented by Perry *et al*. [7].

However, Perry *et al*. [7] also observed that some patients may be asymptomatic and may be picked as an incidental finding on a radiograph. The tendency for orofacial tumors to affect the maxilla and mandible has been well described in the literature [4,7,11]. We recorded more common involvement of the maxilla than the mandible with the left side twice as affected as the right side. The fact that we had a sizable number of patients with Burkitt´s lymphoma may have contributed to this considering the fact that Burkitt´s lymphoma favors the maxilla more than the mandible [14]. While Taiwo *et al*. [11] in their study found the mandible to be more affected than the maxilla. Perry *et al*. [7] on the other hand found almost equal affectation of the maxilla and the mandible with the left and right side also equally affected. Factors that may determine the site of the lesion may include the location of the type of tissue involved as well as the biologic behavior of the lesion. In individuals with skeletal involvement, multilocular radiolucency was the commonest presentation observed. It is worthy of note that sometimes in childhood lesions because histopathological results do not match tumor´s aggressive behavior, treatment may be determined through tumor location, as well as radiographic presentations. A well circumscribed tumor, on radiographic examination may shell out with ease and also with reduced chance of recurrence.

In contrast, tumors with radiographic features that suggest infiltration into surrounding structures may necessitate more aggressive surgical approach in order to ensure complete clearance. Histological diagnosis in our study showed that 30.5% of the tumors were odontogenic while 69.5% were non odontogenic i.e. a ratio of 1:3.3. Burkitt lymphoma which had the highest proportion is non-odontogenic and malignant and was also seen most commonly in the 0-4 years of age category further buttressing the fact that in Africa it is a childhood malignancy [15]. The second most common tumor was the adenomatoid odontogenic tumors which is benign and odontogenic occurred most commonly in the older children (those aged 10-14 years). The two tumor types were however equally distributed by gender. Malaria holoendemicity in our region favors the prevalence of Burkitt´s lymphoma. A finding supported by previous studies in Nigeria [2,11,12]. This study also documented that when the tumors involved both the mandible and maxilla they were most of the time (78.6%) malignant. Cancer has been observed to be a disease of the poor [16] and as documented in the annual “Cancer Facts and Figures 2011” released by the American cancer society, “poverty is a carcinogen” - a cancer causing agent [16,17]. This assertion is supported by the findings that majority of the parents were in social class IV and V which is the lower socio-economic class based on Oyedeji classification [18].

## Conclusion

In our center, paediatric jaw tumors are commonest in male children with the 10-14 years´ age group most commonly affected. The mean time of presentation was 8 months with the maxilla and the mandible on the left side most commonly affected. Burkitt lymphoma and adenomatoid odontogenic tumors were the commonest tumors seen in them; with Burkitt lymphoma occurring more commonly in the younger children and the adenomatoid odontogenic tumors more common in the older children. When the tumor involved both the maxilla and the mandible, the tumor was most likely malignant. We would like to recommend that early presentation at treatment centers must be encouraged since these tumors if presented early can be successfully treated.

### What is known about this topic

- Primary jaw tumors can occur in children;
- They cause tissue damage altering facial growth;
- Tumors in this region can affect psychosocial evolution.

### What this study adds

- There are several histological tumor types that can affect the jaw in children;
- Paediatric jaw tumors are commonest in male children with the 10-14 years´ age group most commonly affected;
- The maxilla and the mandible on the left side is most commonly affected with Burkitt lymphoma and adenomatoid odontogenic tumors being the commonest tumors seen in them.
